# Multidisciplinary Service Utilization Pattern by Advanced Head and Neck Cancer Patients: A Single Institution Study

**DOI:** 10.1155/2012/628578

**Published:** 2012-10-18

**Authors:** Jacqueline C. Junn, Irene A. Kim, Marianna L. Zahurak, Marietta Tan, Katherine Y. Fan, Spencer T. Lake, David Zaboli, Barbara P. Messing, Karen Ulmer, Karen B. Harrer, Dorothy Gold, Keri L. Ryniak, Eva S. Zinreich, Mei Tang, Marshall A. Levine, Ray G. Blanco, John R. Saunders, Joseph A. Califano, Patrick K. Ha

**Affiliations:** ^1^New York Medical College, Valhalla, NY, USA; ^2^Department of Otolaryngology, University of California, Los Angeles, CA, USA; ^3^Johns Hopkins Department of Oncology Biostatistics, Baltimore, MD, USA; ^4^Johns Hopkins Department of Otolaryngology, Baltimore, MD, USA; ^5^Department of Otolaryngology, Montefiore Medical Center, Bronx, NY, USA; ^6^Milton J. Dance Jr. Head and Neck Center, Baltimore, MD, USA; ^7^Department of Radiation Oncology, GBMC, Baltimore, MD, USA; ^8^Department of Medical Oncology, GBMC, Baltimore, MD, USA; ^9^Johns Hopkins Head and Neck Surgery, GBMC, 1550 Orleans Street, Room 5M06, David H Koch Cancer Research Building, Baltimore, MD 21231, USA

## Abstract

*Purpose*. To analyze the patterns and associations of adjunctive service visits by head and neck cancer patients receiving primary, concurrent chemoradiation therapy. *Methods*. Retrospective chart review of patients receiving adjunctive support during a uniform chemoradiation regimen for stages III-IV head and neck squamous cell carcinoma. Univariate and multivariate models for each outcome were obtained from simple and multivariate linear regression analyses. *Results*. Fifty-two consecutive patients were assessed. Female gender, single marital status, and nonprivate insurance were factors associated with an increased number of social work visits. In a multivariate analysis, female gender and marital status were related to increased social work services. Female gender and stage IV disease were significant for increased nursing visits. In a multivariate analysis for nursing visits, living greater than 20 miles between home and hospital was a negative predictive factor. *Conclusion*. Treatment of advanced stage head and neck cancer with concurrent chemoradiation warrants a multidisciplinary approach. Female gender, single marital status, and stage IV disease were correlated with increased utilization of social work and nursing services. Distance over 20 miles from the center was a negative factor. This information may help guide the treatment team to allocate resources for the comprehensive care of patients.

## 1. Introduction

With approximately 400,000 new cases per year worldwide, squamous cell carcinoma is the most common cancer of the head and neck. Concurrent chemo- and radiation therapy is one well-established treatment option for patients who present with advanced stage disease [1–3]. Frequent side effects from radiation therapy include dysphagia, hoarseness, skin desquamation, xerostomia, and mucositis [4]. In addition to improving survival, functional preservation of the involved structures has become increasingly important [5, 6]. The close relationship between the structures involved and fundamental daily activities adds to the challenge in treating patients with head and neck cancer. Hence, assessing their quality of life and identifying characteristics salient to patients utilizing adjunctive services are especially necessary.

Thus far, relatively few studies have sought to investigate the pattern and features attributed to increased use of adjunctive services. The purpose of this study was to identifydemographic and clinical characteristics contributing to an increase in the management needs of patients with advanced head and neck cancer. We analyzed the patterns of resource utilization (social work, nursing, dietitian services) by head and neck cancer patients who received primary, concurrent chemoradiation therapy and assessed the effects of various patient factors (age, gender, tumor characteristics, smoking status, marital status, insurance status, and distance between home and hospital) on such utilization. This study aims to identify the characteristics of patients who visited these services more frequently to assist physicians in optimizing patient care.

## 2. Materials and Methods

### 2.1. Study Subjects

An institutional review board approved retrospective chart review was performed of fifty-two consecutive patients with previously untreated, non-metastatic stage III or IV squamous cell carcinoma of the oropharynx, hypopharynx, or larynx who underwent primary chemoradiation therapy with curative intent. All patients were treated at the Milton J. Dance Jr. Head and Neck Center at the Greater Baltimore Medical Center between 2007 and 2010. Our study included consecutive patients treated from 2007 to 2010. All patients had a histologic diagnosis of squamous cell carcinoma and were staged according to American Joint Committee on Cancer (AJCC) guidelines. Patients with cancer of the salivary glands, sinuses, or unknown primary sties were excluded, as were patients with recurrent tumors or previous chemotherapy or radiation to the head or neck. All patients received adjunctive support during a uniform chemoradiation regimen. Radiation therapy consisted of hyperfractionated dosing of 125 cGy delivered twice daily, at least six hours apart, five days a week over 28 treatment days with a one week treatment break, for a total of 7000–7500 cGy to the primary tumor site. Chemotherapy consisted of either of two cisplatin based regimens. Regimen A included concomitant cisplatin (12 mg/m^2^/1 h) and 5-fluorouracil (600 mg/m^2^/20 h) on days 1 through 5 and 29 through 33. Regimen B included concomitant cisplatin (30 mg/m^2^/1 h) weekly for 6 cycles. All patients underwent prophylactic placement of feeding gastrostomy tubes prior to initiation of chemoradiation.

Retrospective chart review for visits to social workers, nursing, dietitian, and speech/swallowing staff from 2007 to the present was conducted. After provider visits for each patient were quantified, patient factors were evaluated for a correlation with increased resource utilization.

### 2.2. Statistical Analysis

The major statistical endpoint of this study was the determination of factors affecting resource utilization among advanced head and neck patients. Four endpoints, quantified as the number of visits to specialized services, were considered: social work, speech and swallowing, nursing, and dietitian. The sum of all types of visits, overall utilization, was also analyzed. Patient factors tested for an association with these outcomes included gender, age race, Karnofsky performance score, tumor site, disease stage, smoking status, marital status, type of insurance coverage, and the travel distance between the patient's home and the hospital. Each factor was first tested for an association with the utilization outcomes in univariate linear regression models. The simultaneous effect of two or more factors on utilization was studied using multivariate linear regression models. All *P*-values reported are two-sided and all computations were performed using the Statistical Analysis System.

## 3. Results

In this retrospective study, the study population consisted of 17 patients at disease stage III and 35 patients at stage IV, all with a histologic diagnosis of squamous cell carcinoma and treated with a uniform chemoradiation regimen at the Greater Baltimore Medical Center between 2007 and 2010. Patients were predominantly white (88%), males (88%), with tumors of the oropharynx (73%), as detailed in Table 1. The average age was 58.3 (*sd*⁡ = 8.8) years. The median distance from home to the hospital was 20 miles. Patients visited social workers on average 22 times (*sd*⁡ = 16), dietitians 13 times (*sd*⁡ = 7), nurses 12 times (*sd*⁡ = 6), and speech language pathologists 4 times (*sd*⁡ = 4). Overall, the average number of visits to all services was 50 visits (*sd*⁡ = 23).

In univariate analyses, female gender and single marital status were the factors most strongly associated with increased social work visits, indicated in Table 2. Women visited a social worker an average of 16 more times than men (*P* = 0.02), and single patients had 14 more visits than married patients (*P* = 0.003). Patients covered with private insurance utilized on average 10 fewer social work visits than patients with nonprivate insurance (*P* = 0.04). Living more than 20 miles from the hospital decreased the average number of social work visits by about 9 visits, *P* = 0.06, and stage IV disease marginally increased the use of social work service by about 8 visits (*P* = 0.1).

The average number of nursing visits was increased on average by 9 visits for women (*P* = 0.0001) and by 4 visits for patients with stage IV disease (*P* = 0.02); Table 3. In a multivariate regression, adjusting for gender and stage of disease, patients living greater than 20 miles from the hospital saw nurses fewer times by 3 visits than patients living closer, *P* = 0.04. Adjusting for distance from the hospital, female gender and stage IV disease continued to increase the use of nursing services by 8, *P* = 0.0003, and 3, *P* = 0.06, visits, respectively.

None of the studied factors were significantly associated with an increase in dietitian (Table 4) and speech and swallowing visits (Table 5). However, patients over the age of 60 averaged 3 more visits to a dietitian and approximately 2 more visits for speech and swallowing services.

When the sum of visits to all of the adjunctive service was analyzed, female gender, *P* = 0.01, stage IV disease, *P* = 0.03, single marital status, *P* = 0.03, and living closer than 20 miles to the hospital, *P* = 0.04, were associated with increased utilization (Table 6). In the multivariate analysis (Table 7), the strongest independent factors for overall utilization were gender, stage IV disease, and distance from the center. The average utilization was increased by 22 visits for women, *P* = 0.02, and 13 visits for stage IV patients, *P* = 0.04. Living more than 20 miles from the hospital decreased the average number of overall visits by 15, *P* = 0.01.

## 4. Discussion

Treatment of advanced stage head and neck cancer with concurrent chemoradiation warrants a multidisciplinary approach. The current study set out to establish the patient characteristics that required the additional use of adjunctive services in order to help customize care to fit the patients' needs. It is important to recognize that there are a myriad of different primary chemoradiotherapy treatment regimens, and it is possible that our patients utilized services more due to the fact that they underwent hyperfractionated radiotherapy. In our study, certain patient characteristics such as female gender and stage IV disease were correlated with increased utilization of social work and nursing services. Overall, female gender, stage IV disease, and single marital status were found to be significant features in assessing their likelihood of utilizing adjunctive services. On the other hand, distance over 20 miles from the center was a negative predictive factor in the use of these resources as might be expected.

From previous studies, marital status has been shown to be a favorable prognostic factor in recurrence and survival [7–9]. Married individuals, or people living with their significant other, had a lower mortality risk while unmarried patients tended to present with late stage disease [10]. Unmarried patients were also more likely to be left untreated. Furthermore, unmarried patients showed an increased risk of recurrence and mortality compared to their married counterparts, which could be attributed to differences in health-related behavior and social support [7]. Even though previous studies showed that unmarried patients utilized healthcare services less frequently, our analysis showed that single marital status was correlated with increased visits for social work services. One could postulate that the increased use of social work resources by single patients could be due to decreased social support at home. Because social support has been shown to decrease stress in patients [11], it is important to encourage unmarried head and neck cancer patients to seek social support through adjunctive services.

From our study, a more advanced disease stage and female gender predisposed them to use resources more frequently. A high tumor stage has been shown to predict higher distress in patients [12] and to indicate a need for more extensive follow-up to improve survival [13]. Intuitively, patients with advanced stage of disease will need additional care. A retrospective study by Gourin et al. identified factors leading to attrition in a long-term quality of life survey [14]. Through their study, they concluded that patients with advanced tumor stage, recurrent disease, or comorbidities tended to participate more in quality of life analysis. In line with Gourin et al.'s findings, patients with stage IV disease in our study tended to utilize adjunctive resources more frequently. Previous studies have also shown that women use health care services more than men [15, 16]. Similar to those studies, our study showed that female patients utilized adjunctive resources more frequently than male patients.

Another study looked at clinical variables including different means of communications, modes of dietary consumption, pain scale, employment status, and aesthetics to establish scale scores that translate to clinical significance [17]. Our study did not show a marked increase in visits to dietitians regardless of their age, gender, marital status, or the KPS score. A study by Terrell et al. compiled clinical, health behavioral, and demographic variables to determine variables that could be used to predict quality of life for head and neck cancer patients [18]. From their study, characteristics such as the presence of feeding tube, comorbidities, and tracheotomy tube showed negative effects on patients' perception on quality of life. Hence, future patient treatment modalities should consider these findings to improve patient care.

Finally, a study by Gill et al. compared outcome priorities among patients, their companions, and the multidisciplinary medical team [19]. In their study, when the three parties showed unified agreement in outcome priorities, it was correlated with lower post-treatment regret. They concluded that their ability to come to a strong agreement in weighing factors important to patients resulted from having support from their companions and the members of the multidisciplinary team. Therefore, it is ever more important to identify patients who are likely to utilize adjunctive source in order to guide them in the decision making process.

While it is important to remain vigilant about patient features that increase adjunctive resource usage, it is just as crucial to be cognizant of those who utilize these resources sparingly. Although females used these services more frequently, the majority of our patient population consisted of male patients. A practical question in this setting would be to inquire about what could be done to ensure that male patients receive all of the support needed, even when they may themselves minimize their problems. 

## 5. Conclusions

Treatment of advanced stage head and neck cancer with concurrent chemoradiation warrants a multidisciplinary approach. Our study showed that female gender, single marital status, and stage IV disease were correlated with increased utilization of social work and nursing services. Distance over 20 miles from the center was a negative factor in the use of these resources. Although a single institutional experience cannot encapsulate all the salient factors contributing to increased need for adjunctive services for head and neck cancer patients, the information gathered from our study may help guide the treatment team in directing efforts towards patients with predetermined, specific factors. Albeit a limited study, our study is important in initiating the awareness amongst healthcare personnel in identifying patients with increased likelihood of utilizing adjunctive services prior to treatment. Adapting such practice could help minimize psychological and physical risks associated with advanced head and neck cancer, which could lead to better quality of life for these patients. This information may help guide the treatment team in directing efforts and allocating resources for the comprehensive care of patients with advanced head and neck cancer.

## Figures and Tables

**Table 1:** Patient demographics.

Characteristics	Number
Number of patients	52
Mean age	58.3
Race	
Caucasian	46 (88%)
African American	6 (12%)
Gender	
Male	46 (88%)
Female	6 (12%)
Marital status	
Married	37 (71%)
Single	15 (23%)
KPS score*	
70–80	8 (16%)
90	29 (57%)
100	14 (27%)
Smoking status	
Never smoker	15 (29%)
Ever smoker	23 (44%)
Current smoker	14 (27%)
Tumor site*	
Oropharynx	37 (73%)
Nonoropharynx	14 (27%)
Cancer stage*	
III	17 (33%)
IV	34 (67%)

*One unaccounted data point.

KPS: Karnofsky performance status.

**Table 2:** Social work simple regression model.

Factor	Mean visits (95% CI)	*P* value
Sex		
Male	19.8 (15.2–24.2)	0.02
Female	35.7 (19.7–51.6)	
Age (years)		
≥60	19.9 (14.1–25.7)	0.5
<60	22.9 (16.1–29.8)	
Race		
White	20.7 (15.9–25.5)	0.23
African American	29.2 (12.0–46.3)	
KPS score		
High (90 or 100)	20.3 (15.6–25.1)	0.17
Low (70 or 80)	28.9 (13.8–44.0)	
Tumor site		
Nonoropharynx	20.9 (13.6–28.3)	0.85
Oropharynx	21.9 (16.2–27.7)	
Disease stage		
Stage III	16.3 (12.0–20.6)	0.01
Stage IV	24.3 (17.9–30.6)	
Smoking status		
Never smoker	19.8 (14.7–24.9)	
Ever smoker	22.1 (13.0–31.3)	0.67
Current smoker	22.9 (15.2–30.5)	0.62
Marital status		
Married	17.5 (14.2–20.8)	0.003
Single	31.9 (19.0–44.9)	
Insurance		
Nonprivate	29.1	0.04
Private	18.9	
Distance		
<20 miles	25.9	0.06
≥20 miles	17.4	

KPS: Karnofsky performance status.

**Table 3:** Nursing visits: simple linear regression model.

Factor	Mean visits (95% CI)	*P* value
Sex		
Male	11.4 (10.0–12.8)	0.0001
Female	20.7 (13.7–27.6)	
Age (years)		
≥60	11.9 (9.7–14.0)	0.52
<60	12.9 (10.6–15.2)	
Race		
White	12.2 (10.5–14.0)	0.44
African American	14.2 (10.8–17.5)	
KPS score		
High (90 or 100)	12.3 (10.5–14.1)	0.72
Low (70 or 80)	13.1 (9.2–17.0)	
Tumor site		
Nonoropharynx	12.6 (9.4–15.9)	0.89
Oropharynx	12.4 (10.5–14.3)	
Disease stage		
Stage III	9.9 (7.8–12.0)	0.02
Stage IV	13.7 (11.7–15.8)	
Smoking status		
Never smoker	12.5 (9.2–15.9)	
Ever smoker	11.4 (9.6–13.1)	0.53
Current smoker	14.2 (9.9–18.5)	0.43
Marital status		
Married	12.1 (10.0–14.1)	0.42
Single	13.5 (10.8–16.1)	
Insurance		
Nonprivate	13	0.68
Private	12.3	
Distance		
<20 miles	13.7	0.13
≥20 miles	11.3	

KPS: Karnofsky performance status.

**Table 4:** Dietician services: simple linear regression model.

Factor	Mean visits (95% CI)	*P* value
Sex		
Male	12.7 (10.8–14.5)	0.68
Female	13.8 (3.6–24.1)	
Age (years)		
≥60	14.6 (11.8–17.3)	0.09
<60	11.5 (9.0–13.9)	
Race		
White	12.8 (10.8–14.9)	0.91
African American	12.5 (8.7–16.3)	
KPS score		
High (90 or 100)	12.9 (10.9–15.0)	0.72
Low (70 or 80)	12 (6.6–17.4)	
Tumor site		
Nonoropharynx	13.4 (9.3–17.6)	0.67
Oropharynx	12.6 (10.4–14.7)	
Disease stage		
Stage III	11.7 (8.6–14.7)	0.39
Stage IV	13.3 (11.0–15.7)	
Smoking status		
Never smoker	14.5 (10.9–18.2)	
Ever smoker	12.7 (10.2–15.2)	0.4
Current smoker	11.1 (6.5–15.6)	0.16
Marital status		
Married	12.7 (10.5–14.9)	0.88
Single	13 (9.1–16.9)	
Insurance		
Nonprivate	13.4	0.67
Private	12.6	
Distance		
<20 miles	13.7	0.35
≥20 miles	11.9	

KPS: Karnofsky performance status.

**Table 5:** Speech and swallowing: simple linear regression model.

Factor	Mean visits (95% CI)	*P* value
Sex		
Male	3.5 (2.5–4.6)	0.84
Female	3.8 (−0.3–8.0)	
Age (years)		
≥60	4.5 (2.3–6.6)	0.12
<60	2.9 (2.1–3.7)	
Race		
White	3.8 (2.7–4.9)	0.21
African American	1.8 (0.3–3.4)	
KPS score		
High (90 or 100)	3.7 (2.6–4.8)	0.5
Low (70 or 80)	2.8 (0.3–5.2)	
Tumor site		
Nonoropharynx	3.4 (1.7–5.2)	0.88
Oropharynx	3.6 (2.4–4.9)	
Disease stage		
Stage III	2.5 (1.1–3.8)	0.13
Stage IV	4.1 (2.7–5.4)	
Smoking status		
Never smoker	4.8 (2.0–7.6)	
Ever smoker	3.4 (2.1–4.6)	0.22
Current smoker	2.6 (1.2–4.0)	0.1
Marital status		
Married	3.8 (2.4–5.1)	0.54
Single	3.1 (1.8–4.4)	
Insurance		
Nonprivate	4.9	0.11
Private	3.1	
Distance		
<20 miles	3.9	0.52
≥20 miles	3.2	

KPS: Karnofsky performance status.

**Table 6:** Combined services: simple linear regression model.

Factor	Mean visits (95% CI)	*P* value
Sex		
Male	47.4 (40.9–53.9)	0.01
Female	74 (51.1–96.9)	
Age (years)		
≥60	50.8 (41.4–60.2)	0.93
<60	50.2 (40.8–59.6)	
Race		
White	49.5 (42.3–56.7)	0.43
African American	57.7 (40.9–74.4)	
KPS score		
High (90 or 100)	49.3 (42.2–56.4)	0.41
Low (70 or 80)	56.8 (36.1–77.4)	
Tumor site		
Nonoropharynx	50.4 (36.2–64.6)	1
Oropharynx	50.5 (42.8–58.1)	
Disease stage		
Stage III	40.3 (31.5–49.1)	0.03
Stage IV	55.4 (46.9–63.9)	
Smoking status		
Never smoker	51.7 (41.4–62.0)	
Ever smoker	49.5 (38.2–60.9)	0.79
Current smoker	50.7 (36.5–65.0)	0.91
Marital status		
Married	46 (39.5–52.5)	0.03
Single	61.5 (45.4–77.5)	
Insurance		
Nonprivate	60.4	0.06
Private	46.8	
Distance		
<20 miles	57.1	0.04
≥20 miles	43.8	

KPS: Karnofsky performance status.

**Table 7:** Multivariate regression model.

Factor	Estimate (95% CI)	*P* value
Social work		
Intercept	15.9 (11.0–20.7)	
Female	15.1 (2.7–27.6)	0.02
Single	14.1 (5.3–22.9)	0.002
Nursing visit		
Intercept	11 (8.5–13.5)	
Female	8.3 (4.0–12.5)	0.0003
Stage IV	2.8 (−0.11–5.66)	0.06
Distance (>20 miles)	−2.7 [(−5.33)–(−0.08)]	0.04
Combined visit		
Intercept	46.4 (35.4–57.4)	
Female	21.7 (3.27–40.1)	0.02
Stage IV cancer	13.3 (0.7–26.0)	0.04
Distance (>20 miles)	−14.8 [(−26.32)–(−3.37)]	0.01
